# Is Intravenous Magnesium Sulfate Beneficial in Children with Acute Asthma Exacerbation and Acute Bronchiolitis? A Retrospective Cohort Study

**DOI:** 10.3390/children13050704

**Published:** 2026-05-20

**Authors:** Yasin Selcuk Yardibi, Begum Baris Cetinkaya, Zeynep Tobcu, Sevim Orum, Dondu Ulker Ustebay, Sefer Ustebay

**Affiliations:** 1 Department of Pediatrics, Faculty of Medicine, Bandırma Onyedi Eylül University, 10200 Bandırma, Türkiye; yyardibi@bandirma.edu.tr (Y.S.Y.); bcetinkaya@bandirma.edu.tr (B.B.C.); ztobcu@bandirma.edu.tr (Z.T.); sevimorum@bandirma.edu.tr (S.O.); 2 Department of Pediatric Neurology, Faculty of Medicine, Bandırma Onyedi Eylül University, 10200 Bandırma, Türkiye; dustebay@bandirma.edu.tr

**Keywords:** acute asthma exacerbation, acute bronchiolitis, intravenous magnesium sulfate, respiratory distress, pediatric emergency care, bronchodilator therapy

## Abstract

**Highlights:**

**What are the main findings?**

- Intravenous magnesium sulfate improved oxygen saturation in children with acute asthma exacerbations.
- It reduced tachypnea in children with acute asthma exacerbations.

**What are the implications of the main findings?**

- Early administration of IV magnesium sulfate is safe and effective for children with acute asthma exacerbations.
- It may serve as a useful supportive treatment for children with severe respiratory distress.

**Abstract:**

Background/Objectives: Asthma, one of the most common chronic diseases in childhood, and acute bronchiolitis, a leading cause of hospitalization in early childhood, remain significant contributors to morbidity and mortality. Methods: This retrospective cohort study evaluated the efficacy and safety of intravenous magnesium sulfate (IV MgSO_4_) as a secondary treatment in pediatric patients with acute asthma exacerbation unresponsive to first-line therapy and in patients with acute bronchiolitis unresponsive to supportive care. A total of 450 patients aged 6 months to 18 years, including 252 with acute asthma exacerbation and 198 with acute bronchiolitis, were included. Results: Significant improvements in peripheral capillary oxygen saturation were observed after IV MgSO_4_ administration in both groups (*p* < 0.001). In the acute asthma exacerbation group, IV MgSO_4_ also significantly reduced tachypnea compared to the acute bronchiolitis group (*p* < 0.001). No adverse effects related to IV MgSO_4_ were observed. Conclusions: These findings suggest that IV MgSO_4_ may be both beneficial and safe as an early secondary treatment in acute asthma exacerbations.

## 1. Introduction

Acute asthma exacerbations and acute bronchiolitis are common pediatric respiratory diseases causing substantial morbidity and health care costs. Asthma accounts for roughly 750,000 emergency department visits and 200,000 hospitalizations annually in the United States, while acute bronchiolitis, primarily of viral origin, is a leading cause of respiratory distress and hospitalization in children under 24 months [1,2,3,4,5].

**Figure 1:**
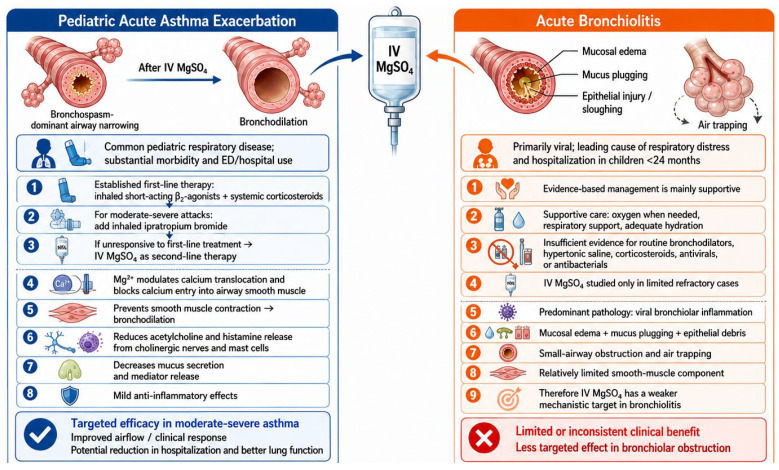
Differential rationale and response to early intravenous magnesium sulfate in pediatric acute asthma exacerbation and acute bronchiolitis.

Magnesium plays a key role in calcium translocation across cell membranes and serves as a cofactor in numerous enzymatic reactions. It blocks calcium channels linked to N-methyl-D-aspartate (NMDA) receptors, preventing calcium entry into smooth muscle cells, thereby inhibiting contraction and producing a bronchodilator effect. Magnesium also reduces mucus secretion by inhibiting acetylcholine and histamine release from cholinergic nerves and mast cells and exhibits anti-inflammatory properties [4] (Figure 1).

First-line treatment of pediatric acute asthma exacerbations is well established and evidence-based. Initial management includes inhaled short-acting beta-2 agonists and systemic corticosteroids. In children and adolescents with moderate to severe exacerbations, inhaled ipratropium bromide is recommended in addition to beta-2 agonists as standard therapy [6,7].

Studies show that intravenous magnesium sulfate (IV MgSO_4_), a second-line therapy for children unresponsive to first-line treatment, is effective in managing moderate to severe asthma exacerbations [1,8,9,10]. Although some systematic reviews suggest that IV MgSO_4_ as a second-line therapy in pediatric acute asthma exacerbations may reduce hospitalization and improve lung function, a recent Cochrane review highlights limited evidence regarding its efficacy and safety due to small sample sizes and few studies [11,12,13,14].

Acute bronchiolitis usually follows a benign course, with most patients managed as outpatients; however, some cases may deteriorate rapidly, requiring respiratory support and admission to a pediatric intensive care unit. Current evidence-based management is primarily supportive, including oxygen therapy when needed, respiratory support, and adequate hydration. There is insufficient evidence to support other interventions, such as inhaled bronchodilators, hypertonic saline, corticosteroids, or antiviral and antibacterial agents [2,3]. The use of IV MgSO_4_ in pediatric patients with acute bronchiolitis unresponsive to supportive care has been evaluated in a limited number of studies [15,16,17].

The primary objective of this study was to assess the efficacy and safety of early IV MgSO_4_ as a second-line treatment in children with acute asthma exacerbations. The secondary objective was to evaluate its effects on clinical outcomes in moderate to severe acute bronchiolitis refractory to supportive treatment. The rationale for including both distinct disease entities in the same study was to directly contrast their differential clinical responses to IV MgSO_4_, thereby highlighting the targeted efficacy of the treatment in asthma compared to its limited benefit in bronchiolar obstruction.

## 2. Materials and Methods

### 2.1. Study Design and Setting

This retrospective observational cohort study was conducted in the emergency department of a tertiary care university hospital between 2021 and 2024.

### 2.2. Patient Selection

The medical records of a total of 1326 patients presenting with acute asthma exacerbation (n = 462) or acute bronchiolitis (n = 864) were retrospectively reviewed. Among these, 252 pediatric patients with acute asthma exacerbation who did not respond to first-line treatment (including inhaled short-acting beta-2 agonists and systemic corticosteroids) within the first two hours and subsequently received intravenous magnesium sulfate (MgSO_4_) as second-line therapy were included. Additionally, 198 patients with acute bronchiolitis who showed an inadequate response to supportive care and were treated with IV MgSO_4_ were also included. An inadequate response was defined as persistent hypoxemia, ongoing severe tachypnea, or lack of clinical improvement in respiratory distress despite standard oxygen therapy and adequate hydration. Patients diagnosed with asthma who had received initial treatment at other centers prior to presentation were excluded. The diagnosis of acute asthma exacerbation and the management of first-line treatment were defined in accordance with international guidelines (e.g., Global Initiative for Asthma—GINA). For the bronchiolitis group, only previously healthy patients with no history of prematurity were included. Based on these criteria, a total of 450 patients aged between 6 months and 18 years were included in the final analysis. IV MgSO_4_ was administered at a dose of 40 mg/kg (maximum 2 g). In patients who required a second dose due to ongoing respiratory distress, the time interval between the two doses was 1 h. The use of IV MgSO_4_ for the management of respiratory distress increased over the study period, with 26 patients (5.8%) in 2021, 96 patients (21.3%) in 2022, 149 patients (33.1%) in 2023, and 179 patients (39.8%) in 2024. This trend is consistent with previously reported literature [18,19,20,21,22,23].

### 2.3. Data Collection

Demographic characteristics, including age and gender, were recorded for all patients. Baseline assessments included blood gas parameters, peripheral capillary oxygen saturation (SpO_2_), heart rate, respiratory rate, and body temperature. Serum magnesium, sodium, and C-reactive protein (CRP) levels were measured prior to treatment. SpO_2_ and respiratory rate were re-evaluated after treatment. Pulmonary findings, including bronchospasm and auscultation findings, were assessed before and after treatment. Pre-treatment chest radiographs were evaluated for the presence of lung infiltration. Nasal swab polymerase chain reaction results for respiratory syncytial virus (RSV), influenza, and Severe Acute Respiratory Syndrome Coronavirus 2 (SARS-CoV-2) were analyzed to determine viral etiology.

### 2.4. Outcome Measures

Clinical improvement was primarily assessed based on changes in lung auscultation findings, as documented in medical records before and 1 h after the completion of IV MgSO_4_ treatment. Peripheral oxygen saturation levels measured before and 1 h after the treatment were analyzed statistically to evaluate treatment response. Additionally, respiratory rate (breaths per minute) prior to and 1 h following the infusion was compared to assess clinical improvement. Furthermore, patients were closely monitored during and after the infusion for potential adverse effects specific to IV MgSO_4_, including hypotension, bradycardia, flushing, and hypotonia. Patients were also evaluated according to the number of IV MgSO_4_ administrations, and its association with clinical response was analyzed. Hospitalization status was not included as an outcome measure, as all patients were admitted either to the pediatric ward or the intensive care unit.

### 2.5. Ethical Approval

The study was approved by the Non-Interventional Research Ethics Committee of Bandırma Onyedi Eylül University Faculty of Medicine (approval no. 2025-03-06; meeting date: 12 May 2025; decision no. 2025-6). The study was conducted in accordance with the Declaration of Helsinki.

### 2.6. Statistical Analysis

Data were summarized using descriptive statistics. Continuous variables were expressed as mean ± standard deviation or median (minimum–maximum), depending on the distribution. Categorical variables were presented as numbers and percentages. Normality of distribution was assessed using the Shapiro–Wilk test. The Mann–Whitney U test was used to compare continuous variables between two independent groups when parametric assumptions were not met. The Wilcoxon signed-rank test was used for paired comparisons (pre- and post-treatment measurements). Associations between categorical variables were evaluated using the chi-square (χ^2^) test. Statistical analyses were performed using MedCalc^®^ Statistical Software version 22.009 (MedCalc Software Ltd., Ostend, Belgium), and a *p*-value of <0.05 was considered statistically significant.

## 3. Results

Among the 252 children with acute asthma exacerbation, 88 (35%) were female and 164 (65%) were male. Among the 198 children with moderate-to-severe acute bronchiolitis, 59 (30%) were female and 139 (70%) were male. The mean age was 88.0 ± 43.3 months in the asthma group and 14.2 ± 5.1 months in the bronchiolitis group (Table 1).

In the acute asthma exacerbation group, blood gas analysis showed normal oxygenation in 52% of patients, mild hypoxemia in 36.1%, moderate hypoxemia in 6.7%, and severe hypoxemia in 5.2%. In the moderate to severe bronchiolitis group, normal oxygenation was observed in 45.5% of patients, mild hypoxemia in 38.9%, moderate hypoxemia in 11.1%, and severe hypoxemia in 4.5%. Hyponatremia was observed in 29.8% of patients across both groups. Low serum magnesium levels were detected in 4.4% of patients with acute asthma exacerbation and 3.5% of patients with acute bronchiolitis (Table 2). Among patients with acute asthma exacerbation, 68 had mild hyponatremia (130–135 mEq/L), and 7 had moderate hyponatremia (125–130 mEq/L). In the acute bronchiolitis group, 50 out of 59 patients had mild hyponatremia, and 9 had moderate hyponatremia. No cases of severe hyponatremia (<125 mEq/L) were observed in either group (Table 2).

Intravenous MgSO_4_ was administered as a single dose in 194 (77%) patients with acute asthma exacerbation and as two doses in 58 (23%) patients. In the bronchiolitis group, 160 (80.8%) patients received a single dose and 38 (19.2%) received two doses. Improvement in lung auscultation findings was observed in 66.7% (n = 168) of asthma patients and 49.5% (n = 96) of bronchiolitis patients following IV MgSO_4_ administration (Table 2).

In both groups, a significant increase in SpO_2_ levels was observed following IV MgSO_4_ administration (*p* < 0.001) (Table 3). After treatment, improvement in respiratory rate was observed in 83.3% (n = 210) of asthma patients and 49.5% (n = 96) of bronchiolitis patients (Table 3).

In the asthma group, the number of IV MgSO_4_ doses did not have a significant effect on the reduction in respiratory rate (*p* = 0.255). However, a significantly greater reduction in tachypnea was observed in asthma patients compared to those with bronchiolitis, depending on the timing of IV MgSO_4_ administration (*p* < 0.001) (Table 4).

## 4. Discussion

The literature demonstrates considerable variability in secondary treatment approaches for pediatric acute asthma exacerbations, including regional differences in the use of intravenous albuterol/salbutamol, intravenous magnesium sulfate (IV MgSO_4_), and aminophylline [1,6,24,25,26]. Multicenter surveys of pediatric emergency physicians in the UK, Ireland, Australia, and New Zealand have reported differing preferences, reflecting regional practice patterns. Consistent with previous reports [18,19,20,21,22,23], the use of IV MgSO_4_ as a second-line treatment for acute asthma exacerbations has increased over the years in our single-center cohort, highlighting its growing role in pediatric asthma management.

In our study, early administration of IV MgSO_4_ as a second-line treatment for acute asthma exacerbation was associated with clinical improvement in approximately two-thirds of patients. Improvements were observed in bronchospasm, lung auscultation findings, oxygenation, and tachypnea. These results are consistent with prior studies indicating that early IV MgSO_4_ administration is safe and effective in pediatric asthma exacerbations [11,20,22]. In contrast, children with moderate-to-severe acute bronchiolitis exhibited a more limited response to IV MgSO_4_, with no significant effect on bronchospasm or lung auscultation findings, which aligns with previous reports [15,16,17]. While a modest improvement in oxygenation was noted, the overall clinical benefit appeared less pronounced compared with asthma exacerbations.

Although no significant difference was observed in respiratory rate reduction according to the number of IV MgSO_4_ administrations in children with acute asthma exacerbation, early administration was associated with a greater reduction in tachypnea compared to children with moderate-to-severe bronchiolitis. This suggests that early IV MgSO_4_ may be particularly effective in relieving respiratory distress in acute asthma, but its clinical benefit in bronchiolitis appears limited. The lack of significant improvement in tachypnea in patients with moderate-to-severe bronchiolitis, independent of dosing, is consistent with previous reports showing a lower response to magnesium treatment in bronchiolar obstruction [15,16,17]. All patients were admitted to either the pediatric ward or the intensive care unit following emergency department intervention, which prevented assessment of potential effects after hospital discharge. Importantly, consistent with previous studies, no adverse effects were observed with IV MgSO_4_, supporting its safety in pediatric populations [1].

Epidemiological studies indicate a higher incidence of asthma in males during early childhood (0–9 years) [27], which aligns with the gender distribution observed in our cohort. Viral infections, particularly RSV, have been increasingly linked to bronchiolitis and subsequent preschool wheezing or asthma [28]. In line with previous studies, RSV remained the most common viral cause of bronchiolitis in our cohort, followed by influenza and SARS-CoV-2 [29]. These findings reinforce the central role of RSV in pediatric bronchiolitis and highlight the need for further investigation of SARS-CoV-2 in this context.

Hyponatremia in acute asthma exacerbations and moderate-to-severe bronchiolitis was generally mild to moderate and transient. Although all patients were normovolemic, inappropriate antidiuretic hormone (ADH) secretion may have contributed, but serum and urine osmolality measurements were not available to confirm this mechanism. Other factors, such as hypotonic fluid administration and excessive hydration, may worsen sodium imbalance. While usually asymptomatic, hyponatremia can cause neurological complications in susceptible patients. Careful monitoring of fluid and electrolyte balance is therefore recommended [30,31,32].

Serum magnesium levels have been investigated in relation to asthma, with some studies suggesting a protective effect of higher dietary magnesium intake, while others report no significant differences between patients and controls [33,34]. In our cohort, no significant decrease in serum magnesium levels was detected in children with acute asthma exacerbation or acute bronchiolitis.

This study has several limitations. The retrospective, observational design precluded assessment of the superiority of IV MgSO_4_ over alternative second-line therapies, and the lack of standardized clinical scores limited detailed comparative analyses. Importantly, the absence of a comparator group limits our ability to definitively isolate the specific effect of IV MgSO_4_, as the observed clinical improvements could partially reflect natural disease progression, the delayed effects of ongoing first-line therapies (such as bronchodilators and corticosteroids), or standard supportive care (including oxygen therapy). Reliance on physician-documented lung auscultation findings as a primary outcome is inherently subjective and may introduce observer bias. Moreover, due to the retrospective nature of the study based on emergency department visits, detailed data on atopic history and pulmonary function test results were not routinely available for all patients during the acute attack. Furthermore, data on the impact of IV MgSO_4_ on hospital length of stay could not be evaluated. Additionally, CRP, lung infiltration findings, temperature, and serum sodium and magnesium levels were reported proportionally, preventing correlation with other clinical parameters.

Despite these limitations, our study has several strengths. Unlike most previous studies, our relatively large cohort provides valuable evidence regarding IV MgSO_4_ as a second-line treatment for pediatric acute asthma exacerbation and bronchiolitis. Furthermore, our study is among the few evaluating early IV MgSO_4_ administration, investigating viral etiologies, and reporting serum sodium and magnesium levels, enhancing its contribution to the literature.

## 5. Conclusions

Intravenous magnesium sulfate administration as a second-line treatment in children with acute bronchiolitis who did not respond to supportive therapy did not result in significant clinical improvement, consistent with previous studies. In contrast, early administration of IV MgSO_4_ in children with acute asthma exacerbations was associated with significant improvements in tachypnea, lung auscultation findings, and oxygen saturation. No adverse effects related to IV MgSO_4_ were observed in either patient group.

These findings indicate that early IV MgSO_4_ administration may be both safe and beneficial in pediatric acute asthma exacerbations. Further prospective, controlled studies are needed to confirm its efficacy and safety profile across different pediatric respiratory conditions.

## Figures and Tables

**Table 1:** Demographic data.

		Asthma (n, %)	Bronchiolitis (n, %)
Gender	Male	88 (34.9%)	59 (29.8%)
	Female	164 (65.1%)	139 (70.2%)
Age (months)	Median (min-max)	88.0 ± 43.3	14.2 ± 5.1
	Mean ± SD	73.5 (24–178)	13 (5–29)

**Table 2:** Patient data.

		Asthma (n, %)	Bronchiolitis (n, %)
Nasal swab	RSV (+)	2 (0.8%)	114 (57.6%)
	SARS-CoV-2 (+)	7 (2.8%)	1 (0.5%)
	Influenza (+)	30 (12%)	32 (16.2%)
	Negative	174 (69%)	40 (20.2%)
	No result	37 (14.6%)	4 (2%)
	RSV + SARS-CoV-2	0 (0%)	5 (2.5%)
	RSV + Influenza	1 (0.4%)	2 (1%)
	SARS-CoV-2 + Influenza	1 (0.4%)	0(0%)
Blood gas acid-base status	Normal	107(42.5%)	64 (32.3%)
	Respiratory acidosis	100 (39.7%)	129 (65.2%)
	Respiratory Alkalosis	45 (17.8%)	5 (2.5%)
Blood gas oxygenation	Normal	131 (52%)	90 (45.5%)
	Mild hypoxemia	91 (36.1%)	77 (38.9%)
	Moderate hypoxemia	17 (6.7%)	22 (11.1%)
	Severe hypoxemia	13 (5.2%)	9 (4.5%)
Sodium	Normal	175 (69.4%)	137 (69.2%)
	Hyponatremia	75 (29.8%)	59 (29.8%)
	Hypernatremia	2 (0.8%)	2 (1%)
Magnesium	Normal	241 (95.6%)	191 (96.5%)
	Hypomagnesemia	11 (4.4%)	7 (3.5%)
CRP	Negative	158 (62.7%)	118 (59.6%)
	Positive	94 (37.3%)	80 (40.4%)
Fever	None	193 (76.6%)	66 (33.3%)
	Yes	59 (23.4%)	132 (66.7%)
Heart Rate	Normal	173 (68.7%)	116 (58.6%)
	Tachycardia	79 (31.3%)	82 (41.4%)
Infiltration on chest X-ray	No pathology	176 (69.8%)	163 (82.3%)
	Pathology present	76 (30.2%)	35 (17.7%)
Number of IV MgSO_4_ Applications	1	194 (77%)	160 (80.8%)
	2	58 (23%)	38 (19.2%)
Lung auscultation findings	No response	84 (33.3%)	102 (51.5%)
	Response present	168 (66.7%)	96 (49.5%)

**Table 3:** IV MgSO4 treatment, SpO_2_, and respiratory rate changes.

**Acute Asthma Exacerbation** **SpO_2_**	**Before**	**After**	**Difference**	***p***
Mean ± SD	92.7 ± 2.2	96.2 ± 2.5	3.5 ± 2.2	<0.001
Median (min-max)	93 (84–96)	97 (88–100)	4 (−2–9)	
**Acute Bronchiolitis** **SpO_2_**	**Before**	**After**	**Difference**	***p***
Mean ± SD	92.3 ± 2.1	95.1 ± 2.9	2.9 ± 2.1	<0.001
Median (min-max)	92 (85–96)	96 (88–99)	3 (−3–8)	
**Respiratory rate per minute**	**Acute Asthma Attack** **(n = 252)**		**Acute Bronchiolitis** **(n = 198)**	
Tachypnea subsided	210 (83.3%)		96 (49.5%)	
Tachypnea persists	42 (16.7%)		102 (51.5%)	

**Table 4:** Tachypnea response according to disease group and number of IV MgSO_4_ administrations.

Number of IV MgSO_4_	Disease Group	Clinical Response (−) n (%)	Clinical Response (+) n (%)	Intergroup *p*-Value	Intragroup *p*-Value
First Application	Acute asthma exacerbation	29 (14.9%)	165 (85.1%)	<0.001 ^a^	0.255 ^c^
	Acute bronchiolitis	72 (45.0%)	88 (55.0%)		0.067 ^c^
Second Applications	Acute asthma exacerbation	13 (22.4%)	45 (77.6%)	<0.001 ^b^	
	Acute bronchiolitis	24 (63.2%)	14 (36.8%)		

IV MgSO_4_, intravenous magnesium sulfate. Intergroup *p*-values compare acute asthma exacerbation and acute bronchiolitis within the same IV MgSO_4_ administration sequence. Intragroup *p*-values compare one and two IV MgSO_4_ administrations within each disease group. ^a^ Pearson chi-square test for intergroup comparisons; ^b^ Yates continuity correction; ^c^ Pearson chi-square test for intragroup comparisons.

## Data Availability

The data presented in this study are available upon request from the corresponding author. Data are not publicly available for confidentiality and ethical reasons.

## Abbreviations

The following abbreviations are used in this manuscript:

IV MgSO_4_	İntravenous magnesium sulfate
SpO_2_	Peripheral capillary oxygen saturation
CRP	C-reactive protein
RSV	Respiratory syncytial virus
